# “Immunonutrition” Has Failed to Improve Peritonitis-Induced Septic Shock in Rodents

**DOI:** 10.1371/journal.pone.0147644

**Published:** 2016-01-25

**Authors:** Julie Boisramé-Helms, Grégory Meyer, Su Emmanuelle Degirmenci, Mélanie Burban, Valérie Schini-Kerth, Luc Cynober, Jean-Pascal De Bandt, Michel Hasselmann, Ferhat Meziani

**Affiliations:** 1 EA 7293, Fédération de Médecine Translationnelle de Strasbourg (FMTS), Faculté de médecine, Université de Strasbourg, Strasbourg, France; 2 Service de Réanimation Médicale, Nouvel Hôpital Civil, Hôpitaux Universitaires de Strasbourg, Strasbourg, France; 3 EA 4278, Université d’Avignon, F-84000, Avignon, France; 4 Laboratoire de Biophotonique et Pharmacologie, UMR 7213 CNRS, Faculté de Pharmacie, Université de Strasbourg, Illkirch, France; 5 Département de Chimie Clinique, Hôpital Cochin, Hôpitaux Universitaires Paris Centre, AP-HP, Paris, France; 6 Laboratoire de Biologie de la Nutrition, EA 4466, Faculté de Pharmacie, Université Paris Descartes, Sorbonne Paris Cité, Paris, France; University of Leicester, UNITED KINGDOM

## Abstract

**Background:**

Immunonutrition in sepsis, including n-3 poly-unsaturated fatty acids (PUFAs) or L-arginine supplementation, is a controversial issue that has yielded a great number of studies for the last thirty-five years, and the conclusions regarding the quantity and quality of this support in patients are deceiving. The aim of the present experimental study is to investigate the effects of a pretreatment with enteral nutrition enriched with n-3 PUFAs or L-arginine on vascular dysfunctions, inflammation and oxidative stress during septic shock in rats.

**Design:**

Rats were fed with enteral Peptamen^®^ HN (HN group), Peptamen^®^ AF containing n-3 PUFAs (AF group) or Peptamen^®^ AF enriched with L-arginine (AFA group). On day 4, peritonitis by cecal ligation and puncture (CLP) was performed. Rats were resuscitated (H18) once septic shock was established. After a 4-hour resuscitation, vessels and organs were harvested to assess inflammation, superoxide anion, nitric oxide and prostacyclin levels. Ex-vivo vascular reactivity was also performed.

**Results:**

Compared to CLP-AF or CLP-HN groups, 47.6% of CLP-AFA rats died before the beginning of hemodynamic measurements (*vs*. 8.0% and 20.0% respectively, p<0.05). AF and AFA rats required significantly increased norepinephrine infusion rates to reach the mean arterial pressure objective, compared to CLP-HN rats. Both CLP-AF and CLP-AFA reduced mesenteric resistance arterial contractility, decreased vascular oxidative stress, but increased NF-κB (0.40±0.15 in CLP-AF and 0.69±0.06 in CLP-AFA *vs*. 0.09±0.03 in SHAM rats and 0.30±0.06 in CLP-HN, ß-actin ratio, p<0.05) and pIκB expression (0.60±0.03 in CLP-AF and 0.94±0.15 in CLP-AFA *vs*. 0.04±0.01 in SHAM rats and 0.56±0.07 in CLP-HN, ß-actin ratio, p<0.05), nitric oxide and prostacyclin production in septic rats.

**Conclusions:**

Although n-3 PUFAs or L-arginine supplementation exhibited an antioxidant effect, it worsened the septic shock-induced vascular dysfunction. Furthermore, mortality was higher after L-arginine supplementation.

## Introduction

During the acute stress resulting from critical illness, the intense catabolic state impairs immune functions and alters the inflammatory response. Nutrition may play a major role through complex interferences with pro-inflammatory cytokine synthesis, immune cell regulation and gene expression. The optimization of nutrition as a supportive treatment has thus emerged as a major challenge in septic shock patients. A so-called “immunonutrition”, making profit of the immune-regulatory properties for example of n-3 poly-unsaturated fatty acids (PUFAs) or L-arginine, may prove efficient. N-3 PUFAs modulate the activation of genes involved in the inflammatory process in immune cells, thus reducing the production of pro-inflammatory cytokines and reactive oxygen species, while they increase the synthesis of anti-inflammatory cytokines, and the release of resolvins [1–3]. On the other side, arginine is the precursor of nitric oxide (°NO), which makes it a major factor in many biological functions such as the immune and vascular cell functions [4]. Several studies have shown that plasma arginine levels decrease during sepsis [5–7], and this is associated with mortality [8]. Arginine deficiency may partly account for septic shock-induced vascular dysfunction through a loss of °NO production in the vascular wall, subsequent alteration of endothelium-dependent vasorelaxation [9–11] and cardiovascular dysfunction [12–14]. However, the beneficial effects of arginine supplementation are still debated [7]. Plasma arginine levels are reduced at the early stage of sepsis and the use of immune enhancing diets containing L-arginine was reported to be beneficial in less severe septic patients [15], while deleterious in advanced stages of septic shock [16,17]. A randomized multicenter clinical trial showed that enteral immunonutrition with L-arginine, n-3 PUFAs and micronutrients, increased mortality compared to parenteral nutrition [16]. In this latter study however L-arginine supplementation was not very high. There are few clinical studies in which the diet was solely enriched with L-arginine: in medical intensive care units (ICUs) with low °NO production, an arginine enriched diet tended to improve the SOFA score and no harm effect was noticed [18]. Actually, there is evidence that L-arginine, n-3 PUFAs and anti-oxidants present in large amounts may detrimentally interact [19,20], but there is no evidence that L-arginine is the culprit [21]. Even more so, various animal studies have shown the benefits of arginine supplementation on immunity [22–24]. However, the data are scarce in septic shock models and there is no study on the exclusive L-arginine supplementation during septic shock in humans.

Overall, the results of recent immunonutrition studies in ICU patients remain disappointing [25], providing conflicting data on optimal nutrition in septic shock patients [26–30] and leaving many questions unresolved. Eventually, while some studies reported that enteral supplementation with eicosapentaenoic acid, gamma linolenic acid and antioxidants decreased the mortality rate as well as mechanical ventilation and ICU stay lengths [31], other studies only found a lower incidence of nosocomial pneumonia and organ dysfunction.

The aim of our work was to assess the effects of different nutrition formulations containing n-3 PUFAs and/or L-arginine in a septic shock model in rats.

## Material and Methods

### Animals

The experiments were performed with the approval of the Strasbourg Regional Ethics Committee for Animal Experimentation (CREMEAS, AL/68/75/02/13).

Male Wistar rats weighing 350–450 g from Janvier Labs (Le Genest-Saint-Isle, France) were used. Before the study protocol, the animals were housed separately in temperature and humidity-controlled quarters with constant 12-hour light, 12-hour dark cycles, were provided with standard food (A04 diet; Safe, Villemoisson, France) and water ad libitum.

During all the experiments, humane endpoints were used. Experiments were indeed stopped and the animal euthanized if they met one of the following criteria: signs of suffering not immediately controlled by the anesthetic and/or analgesics injection, weight loss of more than 15% of initial weight during 5 days of experimentation. Rats were euthanized with an intravenous lethal dose of Pentothal (100 mg/kg).

After each anesthesia and surgery, rats are observed until complete awakening, to check there were no sign of shock (ample/depressed respiratory rate and irregular heart rhythm), stress and pain. Pain was then assessed several times a day and if necessary, analgesics injection was performed. The following criteria were sought for the duration of the experiment: 1. Physiological signs: tachycardia, increased respiratory rate; 2. Behavioral signs: defense/escape at manipulation; 3. Appearance: bristling hair, dull, unusual posture.

There was no unexpected death during the experiments.

Animal suffering and distress was minimized after each surgery as described below in this section, by administering analgesics and anesthetics: anesthesia with isoflurane 1–2% (Baxter S.A.S, Maurepas, France); analgesia with subcutaneous sufentanil (Mylan, Pittsburgh PA, USA) (0.1 μg/kg of body weight); subcutaneous injection of 0.1 mL lidocaine 1% (AstraZeneca, Rueil-Malmaison, France) before skin incision.

### Enteral Nutrition

The following are the products for enteral nutrition used in the protocol:

- Peptamen^®^ HN ("HN"), which is a high nitrogen peptide-based diet enteral formulation, recommended for stress patients
- Peptamen^®^ AF (further referred to as "AF"), which is a formulation containing n-3 PUFAs; the product was enriched with an isonitrogenous mixture of non-essential amino acids (4 g nitrogen/L)
- Peptamen^®^ AF enriched with 1.3 g/100 mL of L-arginine (further referred to as "AFA") plus the isonitrogenous mixture of non-essential amino acids (4 g nitrogen/L).

These products were from Nestlé (France), apart from the L-arginine provided by Sigma-Aldrich (Saint-Quentin Fallavier, France).

### Study Protocol (see Fig 1)

After 8 days of acclimation, the rats were fed with different enteral nutrition products during three days before they underwent peritonitis by cecal ligation and puncture (CLP) so as to mimic post-surgical septic shock in patients, and then compared to SHAM rats. Briefly, rats underwent a gastrostomy for enteral feeding under anesthesia. During surgical procedures, rats were anesthetized with isoflurane 1–2% (Baxter S.A.S, Maurepas, France) and analgesia was performed with subcutaneous sufentanil (Mylan, Pittsburgh PA, USA) (0.1 μg/kg of body weight). A subcutaneous injection of 0.1 mL lidocaine 1% (AstraZeneca, Rueil-Malmaison, France) was performed before skin incision. Following left-sided laparotomy, the greater curvature of the stomach was isolated. A silicon catheter was placed inside the stomach and secured by a purse-string suture. The catheter was tunneled subcutaneously to the neck, and attached to a spring coil-swivel mechanism. The laparotomy was closed and the animals were housed in their cages. The rats were then randomly allocated to one of the following groups: SHAM-HN (further referred to as “SHAM” group), CLP-HN, CLP-AF and CLP-AFA. The allocated enteral nutrition (HN, AF, AFA) was administered at identical rates during 4 days, providing 290 kcal/kg/day and 3 g nitrogen/kg/day. Enteral nutrition was not discontinued until rats were sacrificed at the end of the 4^th^ day.

**Fig 1:**
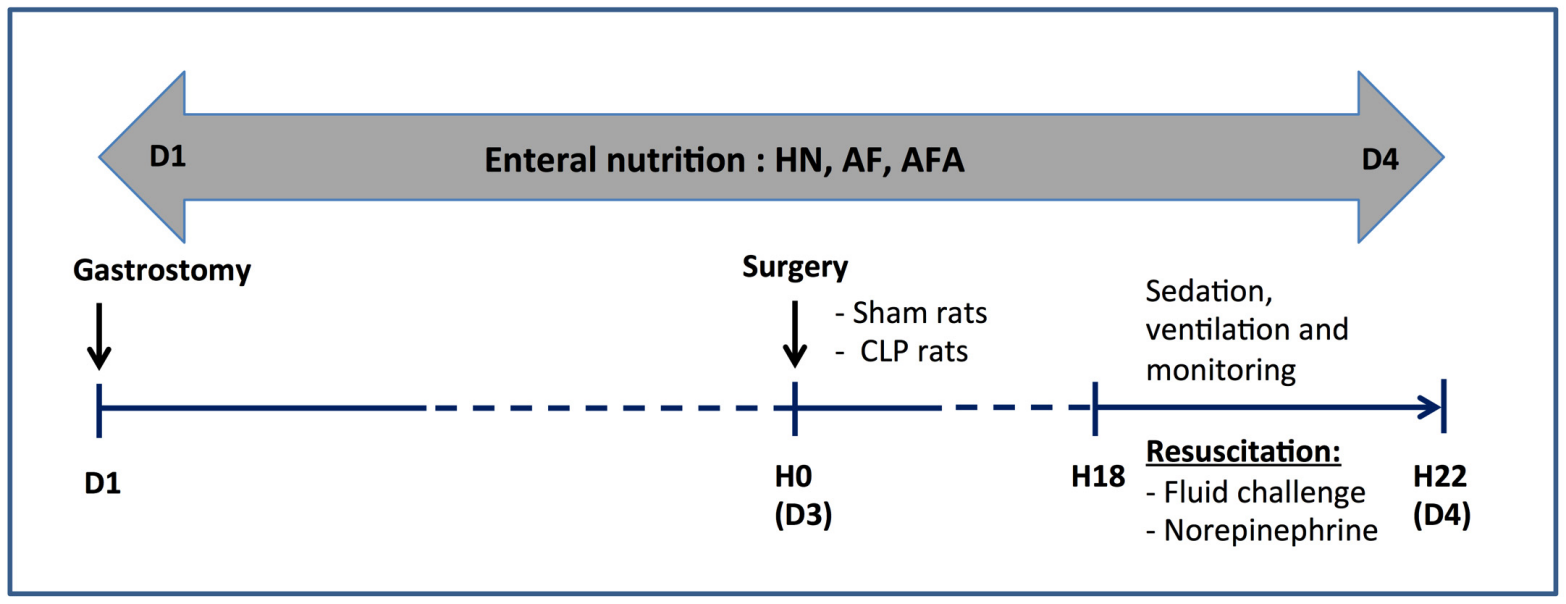
Protocol design. Gastrostomy was performed on day 1 (D1). Rats were then fed with different enteral nutrition products (Peptamen^®^ HN–“HN”, Peptamen^®^ AF–“AF”, Peptamen^®^ AF enriched with arginine–“AFA”) for three days (72 hours) before undergoing peritonitis by cecal ligation and puncture (CLP) or SHAM operation on day 4. Septic shock developed within 18 hours after surgery in the CLP group. Therefore from H18 to H22 after SHAM/CLP surgery, rats were resuscitated using fluid challenge and norepinephrine to target a mean arterial pressure (MAP) value over 100 mmHg. Blood, organs and vessels were collected at the end of the monitoring.

### Cecal Ligation and Puncture Model

On the 4^th^ day (after 72 hours of enteral nutrition), CLP rats underwent cecal ligation and puncture as previously described [32]. The operator was blinded concerning the allocated enteral nutrition. Rats were anesthetized as described above. A 3-cm midline new laparotomy was performed to allow exposure, ligation and puncture of the caecum with an 18-gauge needle. A small amount of feces was extruded, the caecum was returned into the peritoneal cavity and the laparotomy was closed. All rats received a subcutaneous injection of 0,9% NaCl (30 mL/kg body weight). The rats developed septic shock within the 16–20 hours after CLP. SHAM rats underwent a midline laparotomy and cecal exposure without further manipulation. After 18 hours, when CLP rats fulfilled illness criteria (lethargy, piloerection, glassy eyes), all rats were anesthetized, tracheotomized and mechanically ventilated.

The left femoral artery was used to measure mean arterial pressure (MAP) and heart rate (HR) and to collect blood samples. For the CLP group, septic shock was established when the MAP fell below 90 mmHg and lactate increased over 2 mmol/L. In CLP groups, fluid resuscitation was performed with a bolus of 0.9% NaCl (500 μL/10 min) if the MAP was below 100 mmHg; if it remained below 100 mmHg, norepinephrine was infused and increased by step of 0.1 μg/kg/min each 10 minutes to target a MAP above 100 mmHg. After 240 minutes, rats were sacrificed by bloodletting under deep anesthesia; vessels were collected for ex-vivo vascular reactivity and protein expression analysis and electron paramagnetic resonance measurements.

### Vascular Reactivity

Mesenteric and aortic ring vascular reactivity was studied on a wire myograph (Danish Myo Technology, Arhus, Denmark) as previously described [33]. Arteries were bathed in a physiological salt solution (PSS: 119 mM NaCl, 4.7 mM KCl, 14.9 mM NaHCO_3_, 1.2 mM MgSO_4_.7H_2_O, 2.5 mM CaCl_2_, 1.18 mM KH_2_PO_4_, and 5.5 mM glucose under a pH 7.4, PO_2_ 160 mm Hg, PCO_2_ 37 mmHg) continuously bubbled with 95% O_2_ and 5% CO_2_. After an equilibration period of at least 20 min. under the optimal passive tension, two successive contractions in response to the combination of KCl (100 mM) depolarization and serotonin (5-HT; 10 μM) (Sigma-Aldrich) were used to test the maximal contractile capacity of the vessels. After washing, the endothelial function was assessed by testing the relaxing effect of acetylcholine (Ach 1 μM) after precontraction by 1 μM 5-HT. Following a 20-min. washout period, concentration-response curves to 5-HT were elicited by a cumulative administration of the vasoconstrictor agonist (1 nM to 100 μM) to vessels with their endothelium submitted or not to a specific iNOS inhibitor, N-([3-(Aminomethyl)phenyl]methyl) ethanimidamide dihydrochloride (1400W, 100 μM; Sigma-Aldrich), or a specific COX-2 inhibitor, N-(2-cyclohexyloxy-4-itrophenyl)methanesulfonamide (NS-398, 10 μM; Sigma-Aldrich). The inhibitor was added in the bath 30 min. before the addition of 5-HT.

### Electron Paramagnetic Resonance (EPR) Measurements

#### Nitric oxide

The artery samples were incubated for 30 min. in Krebs–Hepes buffer containing bovine serum albumin (20.5 g/L), CaCl_2_ (3 mM), and arginine (0.8 mM). Sodium diethyldithiocarbamate (DETC; 3.6 mg) and FeSO_4_·7H_2_O (2.25 mg) were separately dissolved under nitrogen gas bubbling in 10-mL volumes of ice-cold Krebs–Hepes buffer. These compounds were rapidly mixed to obtain a pale yellow-brown opalescent colloid Fe(DETC)_2_ solution (0.4 mM), which was immediately added to the organs and incubated for 45 min. at 37°C. °NO measurement was performed on a table-top x-band spectrometer Miniscope (Magnettech, MS200, Berlin, Germany). Recordings were made at 77°K, using a Dewar flask. Instrument settings were as follows: microwave power 10 mW, amplitude modulation 1 mT, modulation frequency 100 kHz, Sweep time 60 seconds and scan sequencing on 5.

#### Superoxide anion

The artery samples were allowed to equilibrate in a deferoxamine-chelated Krebs–Hepes solution containing 1 hydroxy-3 methoxycarbonyl 2,2,5,5-tetramethylpyrrolidin (CMH, Noxygen, Germany) (500 μM), deferoxamine (25 μM), and DETC (5 μM) under constant temperature (37°C) for 1 hour. The reaction was stopped by freezing the samples in liquid nitrogen for EPR spectroscopy analysis. Values were expressed as arbitrary units per milligram weight of dried tissue (A/Wd).

### Protein Expression

Phosphorylated I kappa B-alpha (pIκB-α), nuclear factor-kappa B (NF-κB), inducible °NO synthase (iNOS) and cyclo-oxygenases-2 (COX-2) expressions were evaluated in aorta lysates using Western blot. Proteins were separated by electrophoresis in a 10% polyacrylamide gel and transferred onto a PVDF (PolyVinyliDene Fluoride) membrane. Nonspecific binding sites were blocked with 5% skimmed milk in a Tris-Buffered Saline solution with 0.5% Tween for 1 hour at room temperature. Membranes were then incubated overnight at 4°C with primary antibodies directed against pIκB-α (1/500, Euromedex, Souffelweyersheim, France), NF-κB p65 (1/1,000, Ozyme, Saint Quentin, France), COX-2 (1/1,000 Cayman Chemical Co, Ann Arbor, MI), iNOS (1/1,000, Beckton Dickinson), and β-tubulin (1/10,000, Santa Cruz Biotechnology, Heidelberg, Germany). After 3 washes, the membranes were incubated with secondary antibodies (1/5,000 dilution except for anti-β-tubulin that revealed with a 1/40,000 dilution of anti-mouse IgG) for 1 hour at room temperature and signals were revealed by chemoluminescence. The optical density was quantified and expressed relative to β-tubulin (image J).

### Determination of Prostacyclin Production

Prostacyclin (PGI_2_) was measured in the incubation media of arteries harvested from the SHAM, CLP-HN, CLP-AF and CLP-AFA groups, using an ELISA assay kit (Cayman Chemicals, Montluçon, France). The concentrations of free PGI_2_ were expressed in pg/mg of vessel dry weight.

### Statistical Analysis

Mortality was expressed by the Kaplan Meier method and compared with the log-rank test. The inter-group comparison of hemodynamic parameters was performed by ANOVA for repeated measures; the pairwise comparison was made using a Tukey-Kramer adjustment for p values. A nonparametric analysis by a Kruskal Wallis test with a Dunn post hoc test was used for protein, °NO, O_2_°^-^ and prostacyclin analysis. All statistics was performed with Statview™ software (version 5.0, SAS Institute, Cary, NC, USA). All values were presented as mean ± SD for n experiments, with n representing the number of rats. A p value < 0.05 was considered statistically significant.

## Results

### L-Arginine Supplementation Increased Septic Shock-Induced Mortality

**Fig 2:**
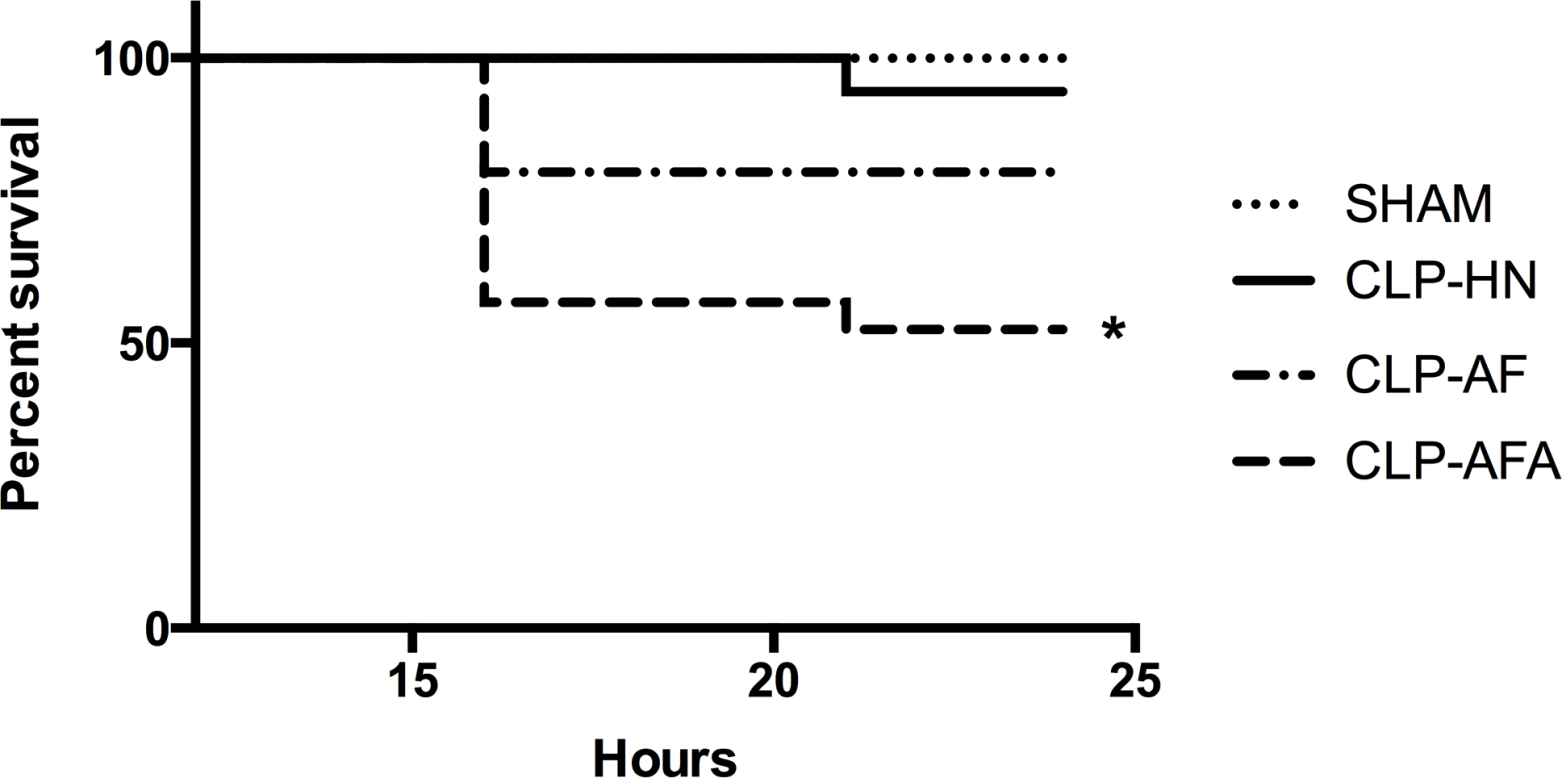
Survival of SHAM and septic rats (CLP-HN, CLP-AF and CLP-AFA) treated with different enteral nutrition formulations: Peptamen^®^ HN (HN), Peptamen^®^ AF (AF), Peptamen^®^ AF enriched with arginine (AFA). * p<0.05 vs. all the other groups. n = 5, 12, 15 and 21 rats in SHAM, CLP-HN, CLP-AF and CLP-AFA groups respectively.

All rats survived during the three-day enteral nutrition after gastrostomy was performed and they did not loose weight. Compared to SHAM rats, all CLP rats were in septic shock 18 hours after peritonitis, as defined above, with norepinephrine infusion requirement and a significant increased plasma lactate level (p<0.05) (Table 1). Compared to CLP-AF and CLP-HN groups, mortality was significantly higher in the CLP-AFA group after peritonitis (p<0.05; Fig 2, S1 Table). Because of an early unexpected mortality in CLP-AF and CLP-AFA groups, after CLP surgery but before hemodynamic measurements (<18^th^ hour), the number of rat has been increased in these 2 groups in order to keep the same number of rats in the 4 groups for hemodynamic measurements (Table 2).

**Table 1:** Characteristics of septic and control rats: clinical records from the onset of septic shock to the end of the experiment and effects of different enteral nutrition formulations.

	SHAM (n = 5)	CLP-HN (n = 12)	CLP-AF (n = 15)	CLP-AFA (n = 21)
**Body Weight (g)**	428 ± 28	389 ± 30	390 ± 22	379 ± 25
**MAP (mmHg)**				
Baseline	123 ± 14	78 ± 9*	80 ± 9*	75 ± 9*
End of experiment	103 ± 19	88 ± 8	91 ± 7	97 ± 8
**HR (bpm)**				
Baseline	338 ± 57	344 ± 22	351 ± 13	339 ± 11
End of experiment	306 ± 54	381 ± 25	349 ± 19	362 ± 19
**CBF (mL/min)**				
Baseline	4.8 ± 1.5	4.5 ± 0.7	6.2 ± 0.8	5.0 ± 0.5
End of experiment	3.8 ± 1.7	3.4 ± 0.5	3.2 ± 0.4	3.2 ± 0.5
**Lactate (mmol/l)**	1.0 ± 0.4	5.2 ± 1.1*	3.3 ± 0.4*	3.4 ± 0.4*

Septic (CLP-HN, CLP-AF, CLP-AFA) and SHAM rats were treated with different enteral nutrition formulations during 4 days. Baseline was recorded 18 hours after CLP or SHAM surgery (performed on the 4^th^ day) and rats were monitored during 4 hours (from the 18^th^ to the 22^nd^ hours). MAP: mean arterial pressure, HR: heart rate, bpm: beat per minute, CBF: carotid blood flow, NE: norepinephrine. Data are expressed as mean ± SD, p<0.05: * *vs*. SHAM.

**Table 2:** Because of an early important mortality after CLP surgery, but before hemodynamic measurement were started (<18^th^ hour), the number of rat has been increased in groups were rats died ≤ 18^th^ hour (H18) after surgery.

**Groups**	**Number of rats/group (+ rats added to compensate deaths <H18)**	**Global mortality before the end of experiment**	**Timing of death**
**SHAM**	5	0 (0%)	0
**CLP-HN**	12	1 (8.0%)	1 rat at H21
**CLP-AF**	12 (+ 3)	3 (20.0%)	3 rats < H18
**CLP-AFA**	12 (+ 9)	10 (47.6%) *	9 rats < H18 and 1 rat at H21

* p < 0.05 versus CLP-HN and CLP-AF.

### PUFA and L-Arginine Supplementations Failed to Improve Septic Shock-Induced Hemodynamic Dysfunction

**Fig 3:**
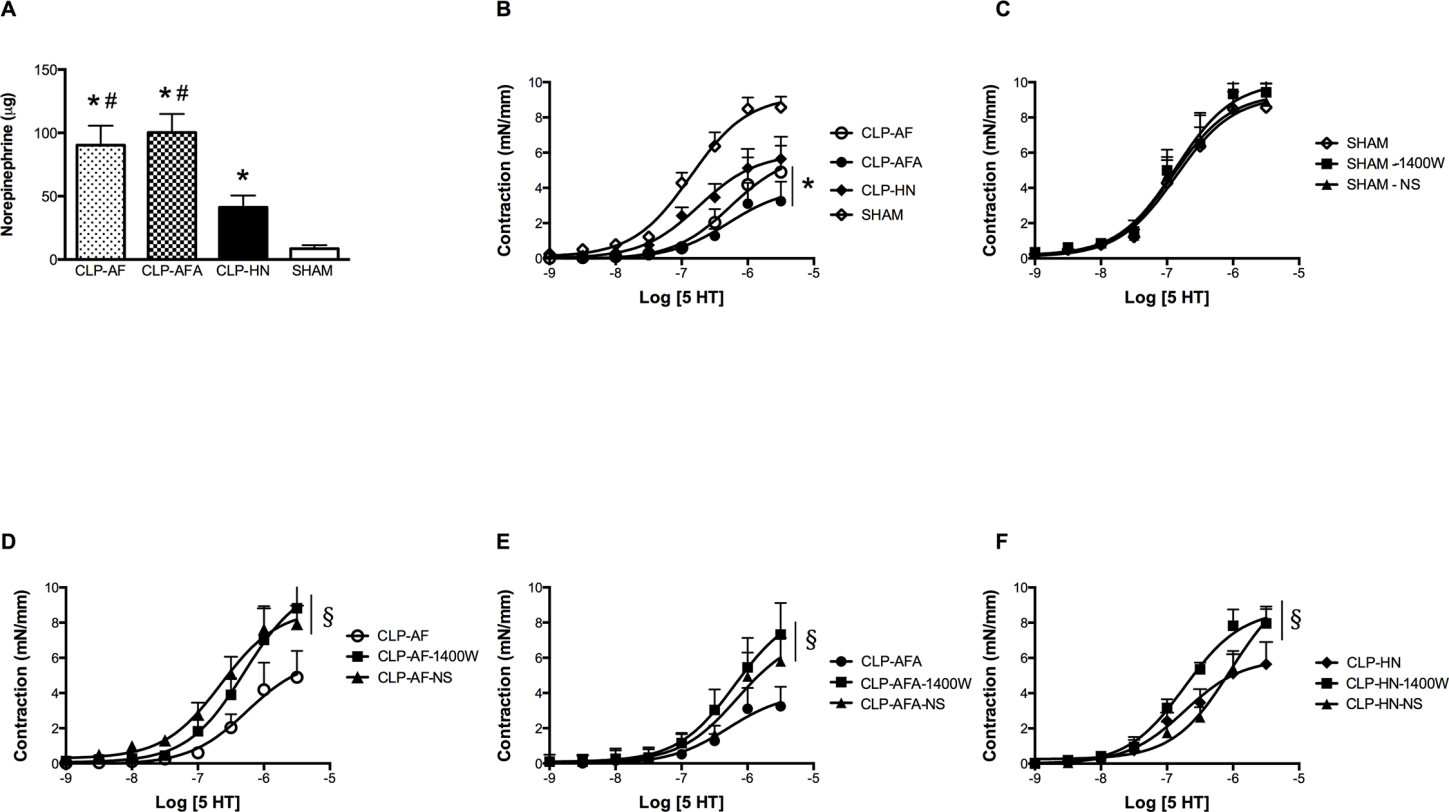
(A-F): A) Cumulative norepinephrine dose needed to reach the mean arterial pressure (MAP) objective in SHAM and septic rats treated with different enteral nutrition formulations: Peptamen^®^ AF (AF), Peptamen^®^ HN (HN), Peptamen^®^ AF enriched with arginine (AFA); * p<0.05 *vs*. SHAM rats, # p<0.05 *vs*. CLP-HN rats. n = 5 for SHAM group and n = 12 for CLP-HN, CLP-AF and CLP-AFA groups. B) *Ex-vivo* vascular contractility of mesenteric resistance arteries of SHAM and septic rats in response to 5-HT, * p<0.05 *vs*. SHAM rats. n = 5 for SHAM group and n = 12 for CLP-HN, CLP-AF and CLP-AFA groups. C-F) Effect of the specific iNOS inhibitor (1400W) and the specific COX-2 inhibitor (NS-398) on 5-HT-dependent contraction in mesenteric resistance arteries in each different group of rats (C = SHAM rats, D = CLP-AF rats, E = CLP-AFA rats, F = CLP-HN rats). § p<0.05 vs. without inhibitor. Results are expressed as mean ± SD. n = 5 for SHAM group and n = 12 for CLP-HN, CLP-AF and CLP-AFA groups.

Mean arterial pressure was successfully maintained at control level since it showed no difference between SHAM and CLP groups (HN, AF, AFA). However, PUFA and L-arginine supplementation significantly increased norepinephrine requirements to reach the targeted MAP, compared to SHAM and CLP-HN groups (85.0 ± 20.2 μg in CLP-AF and 104.0 ± 20.0 μg CLP-AFA groups *versus* 46.0 ± 4.4 μg in CLP-HN and 9.0 ± 1.1 μg in SHAM groups, p<0.05; Fig 3A).

5-HT induced a concentration-dependent increase in the contraction of mesenteric arteries rings (Fig 3B, S2 Table). Compared to the SHAM group, the vascular responses to 5-HT were significantly decreased both in the aortic rings (not shown) and mesenteric arteries harvested from the CLP-AF and CLP-AFA groups, but not in the CLP-HN group (Fig 3B, S2 Table).

In order to investigate the mechanisms involved in this vascular hyporeactivity, the role of °NO and eicosanoids in 5-HT-dependent contraction was evaluated by testing the effect of 1400W and NS-398 respectively. iNOS specific inhibition did not modify the contractile responses to 5-HT in control vessels, but significantly enhanced it in vessels from CLP rats, whatever their enteral nutrition (Fig 3C to 3F, S2 Table). When a COX-2 inhibitor was added, only the hyporeactivity to serotonin of arteries from CLP-AF group was significantly restored to the level of SHAM group, compared to CLP-HN and CLP-AFA groups (Fig 3C to 3F, S2 Table).

### PUFA and L-Arginine Supplementations Differentially Affect Vascular Wall Inflammation

**Fig 4:**
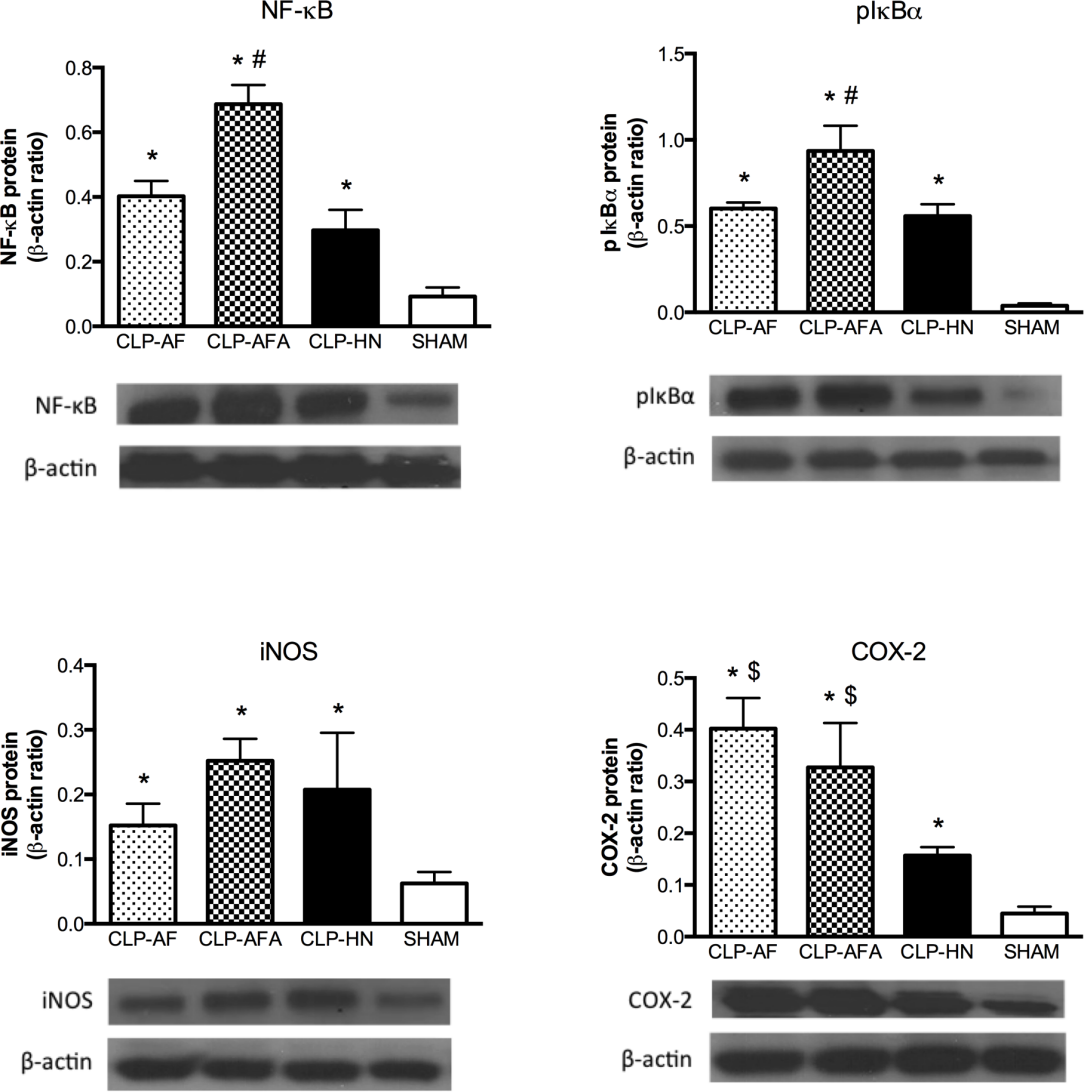
(A-D): A-B) NF-κB p65/RelA expression and pIκB-α content in mesenteric resistance artery (MRA) tissue homogenates from septic and SHAM rats treated with different enteral nutrition formulations: Peptamen^®^ AF (AF), Peptamen^®^ HN (HN), Peptamen^®^ AF enriched arginine (AFA). Densitometry images are representative of six separate blots and mean ± SD densitometry values are expressed in arbitrary units (A.U.) relative to β-tubulin content. Different enteral nutrition formulations were as follows: Peptamen^®^ AF (AF), Peptamen^®^ HN (HN), Peptamen^®^ AF enriched arginine (AFA). * p<0.05 vs. all the other groups, # p<0.05 vs. SHAM rats, & p<0.05 vs. CLP-AF rats, $ <0.05 vs. CLP-HN rats. n = 5 for SHAM group and n = 12 for CLP-HN, CLP-AF and CLP-AFA groups.

After cell stimulation, IκB is phosphorylated, removed and degraded, allowing free NF-κB to induce transcription. CLP significantly increased both NF-κB expression and pIκB content in the aorta, compared to the SHAM group, and significantly more in the CLP-AFA than in the CLP-HN and CLP-AF groups (Fig 4A and 4B, S3 Table).

Compared to SHAM rats, all groups showed a significantly higher expression of iNOS and COX-2 (Fig 4C and 4D, S3 Table). In CLP-AF and CLP-AFA groups, COX-2 expression was significantly higher than in CLP-HN or SHAM (Fig 4D, S3 Table) groups.

### PUFA and L-Arginine Supplementations Differentially Affect Nitric Oxide, Prostacyclin and Superoxide Anion Productions

**Fig 5:**
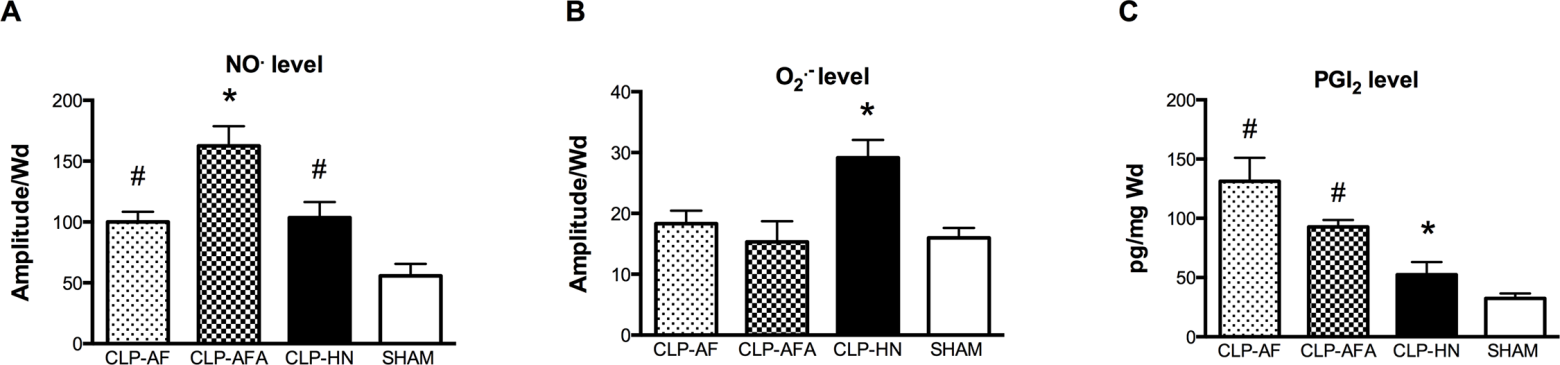
(A-C): O_2_°^-^, NO° and PGI_2_ levels in the aorta from septic and SHAM rats treated with different enteral nutrition formulations: Peptamen^®^ AF (AF), Peptamen^®^ HN (HN), Peptamen^®^ AF enriched arginine (AFA). O_2_°^**-**^ and NO° levels were measured by electron paramagnetic resonance (Values were expressed as arbitrary units per milligram weight of dried tissue (A/Wd)) and PGI_2_ by Elisa. Results are expressed as mean ± SD. * p<0.05 vs. all the other groups, # p<0.05 vs. SHAM rats. n = 5 for SHAM rats and n = 12 for CLP-HN, CLP-AF and CLP-AFA groups.

Compared to the SHAM group, arteries °NO production was significantly higher in all CLP groups, especially in CLP-AFA (Fig 5A, S4 Table) rats. Compared to CLP-HN rats, the two formulations containing PUFAs (AF and AFA) significantly decreased superoxide anion production in arteries from septic rats (Fig 5B, S4 Table), whereas they increased prostacyclin production in arteries (Fig 5C, S4 Table).

## Discussion

With regard to septic shock, immunonutrition has aroused the interest of clinicians for several years. While n-3 PUFAs interfere with immune and inflammatory processes [2], the L-arginine deficit during sepsis [7,34–36] impairs the arginine/°NO pathway, setting off a multiple organ dysfunction syndrome leading to poor clinical outcomes in septic shock. However, there is no clear clinical benefit in supplementing septic patients with either n-3 PUFAs and/or L-arginine [37,38].

In the present study, we have first shown that enteral L-arginine supplementation (AFA) was deleterious in septic rats and increased early mortality, while HN and AF formulations had no significant effect on septic rat mortality. These results are consistent with previous experimental studies. In a canine septic shock model [39], L-arginine infusion was associated with significant worsening in shock status, renal and liver functions, and increased mortality. In humans, there are few data on the sole L-arginine supplementation during septic shock. In his meta-analysis, Heyland [17] concluded that immune-enhancing nutrients, compared to standard diet, may increase mortality in critically ill patients, including septic patients. Bertolini *et al*. [16] later supported these conclusions, showing a significantly increased mortality (44.4% *vs*. 14.3%) in patients with severe sepsis receiving immunonutrition, compared to those fed with standard parenteral nutrition. This excess mortality was attributed to L-arginine, which would enhance °NO production and thus worsen vascular hyporesponsiveness to vasoconstrictor agonists [40]. Surprisingly, supplementing enteral nutrition with n-3 PUFAs (0.8 g/L and 3.6 g/L in HN and AF formulations respectively) and decreasing n-6/n-3 ratio from 6.1 in HN to 2 in AF formulation had no benefit on septic rat survival in our septic shock model.

The excess mortality in septic rats supplemented with L-arginine in our experiments may be due to higher vasopressor needs in this group during septic shock. Although L-arginine infusion in eight patients with septic shock seemed to have little effect on hemodynamic parameters [41], it induced a significant blood pressure and systemic vascular resistance index decrease, with worsened shock status in septic animal models [39,42]. This enhanced vascular dysfunction might be partly explained by an increase in vascular inflammation and/or oxidative and nitrosative stresses, as shown by our results. In the present septic shock model, L-arginine supplementation was indeed responsible for vascular pro-inflammatory and pro-oxidative effects. These results are consistent with previously published data: in a model of mild chronic endotoxemia in pigs, Bruins *et al*. [43] have shown that L-arginine infusion significantly increased whole-body °NO production; the rate of °NO production seemed related to the level of circulating L-arginine, but that was not associated with increased organ perfusion. This suggests that L-arginine was only responsible for minor hemodynamic effects during and after endotoxin challenge. Moreover, like in the CLP-AFA group, we have shown that the enteral AF formulation increased norepinephrine needs in septic rats, with increased prostacyclin production. Compared with the standard enteral HN product, AF is an advanced formulation, mainly enriched with n-3 PUFAs, aiming at reducing pro-inflammatory processes through the modulation of lipid mediator generation, neutrophil metabolism and cytokine response [2]. Like medium- and long-chain triglyceride emulsions, n-3 PUFAs could modulate the inflammation through membrane composition remodeling and subsequent microparticle release alterations [44–46]. Moreover, several studies on hypertension have pointed out that both EPA and DHA promote vasodilation and have an anti-hypertensive effect both in experimental models and in humans, by increasing °NO production and altering eicosanoid synthesis [47]. It was for example shown that n-3 PUFAs enhance large artery endothelium-dependent dilation assessed in flow-mediated dilation in patients with hypercholesterolemia [48]. Our data are consistent with *in vivo* studies showing that n-3 PUFA supplementation increases PGI_2_ generation [49]. Beside these effects, EPA/DHA supplementation could alter the endogenous eicosanoid profile by providing alternative substrates for the arachidonic metabolism by involving the cytochrome P450 pathway. Fischer *et al*. have indeed shown that increasing the n-3 PUFA intake in humans resulted in a large increase of cytochrome P450-dependent epoxy-metabolites of EPA and DHA, showing vasorelaxing effects of n-3 PUFAs [50]. Finally, it has been shown that EPA might inhibit the production of vasoconstrictors such as endothelin-1 in-vitro [51].

In spite of numerous experimental and clinical studies, n-3 PUFA effective potential benefits in septic patients remain controversial [52]. While in some studies the mortality rate, mechanical ventilation and ICU stay lengths are decreased in septic patients supplemented with enteral n-3 PUFAs and antioxidants [31], a randomized multicenter clinical trial recently reported an increased mortality in the immunonutrition group [16]. Heller *et al*. [53] also showed that a parenteral fish oil emulsion increased survival while it decreased infection rates, antimicrobial requirements and length of stay in a dose-dependent manner. However, no difference was observed in another study with regard to inflammation, mortality, length of mechanical ventilation, in septic patients [54]. Barbosa *et al*. [55] and Gultekin *et al*. [56] further confirmed that parenteral fish oil supplementation did not improve the mortality or global outcome of septic patients. Based on our results and the data from the literature, one may therefore question the rationale for n-3 PUFA supplementation during septic shock, compared with standard enteral formulation.

The main limitation of our study lies in the fact that we could not test the effect of an arginine-free enteral diet, since there is no such diet available. Moreover, although rats were resuscitated from septic shock onset in our experiments, they did not receive antibiotics, which would be difficult to adapt according to sanitary condition of rat breeding.

## Conclusion

In this experimental septic shock model, an immunonutrition diet enriched with L-arginine or n-3 PUFAs failed to improve septic shock-induced hemodynamic dysfunction. Moreover, L-arginine supplementation seems to increase septic rat mortality. The need to assess the relevance of these results in septic patients, calls for further studies, and the place for so-called immunonutrition shall be questioned.

## Supporting Information

S1 Table Survival of SHAM and septic rats (CLP-HN, CLP-AF and CLP-AFA) treated with different enteral nutrition formulations: Peptamen® HN (HN), Peptamen® AF (AF), Peptamen® AF enriched with arginine (AFA). (XLSX)

S2 Table Ex-vivo vascular contractility of mesenteric resistance arteries of SHAM and septic rats in response to 5-HT; effects of 1400W and NS-398 on 5-HT-dependent contraction. (XLSX)

S3 Table NF-κB p65/RelA expression and pIκB-α content in mesenteric resistance artery tissue homogenates from septic and SHAM rats treated with different enteral nutrition formulations: Peptamen® AF (AF), Peptamen® HN (HN), Peptamen® AF enriched arginine (AFA). Densitometry values are expressed in arbitrary units (A.U.) relative to β-tubulin content. Different enteral nutrition formulations were as follows: Peptamen® AF (AF), Peptamen® HN (HN), Peptamen® AF enriched arginine (AFA).
(XLSX)

S4 Table O2°-, NO° and PGI2 levels in the aorta from septic and SHAM rats treated with different enteral nutrition formulations: Peptamen® AF (AF), Peptamen® HN (HN), Peptamen® AF enriched arginine (AFA). O2°- and NO° levels were measured by electron paramagnetic resonance (Values were expressed as arbitrary units per milligram weight of dried tissue (A/Wd)) and PGI2 by Elisa.
(XLSX)
